# The Emerging Role of Checkpoint Inhibition in Microsatellite Stable Colorectal Cancer

**DOI:** 10.3390/jpm9010005

**Published:** 2019-01-16

**Authors:** David J. Hermel, Darren Sigal

**Affiliations:** 1 Department of Medicine, University of Southern California, 2020 Zonal Avenue, Los Angeles, CA 90033, USA; david.hermel@med.usc.edu; 2 Division of Hematology/Oncology, Scripps Clinic, 10666 North Torrey Pines Road, La Jolla, CA 92037, USA

**Keywords:** immunotherapy, microsatellite stable, colorectal cancer, immune checkpoint inhibitors

## Abstract

Checkpoint inhibitor therapy has introduced a revolution in contemporary anticancer therapy. It has led to dramatic improvements in patient outcomes and has spawned tremendous research into novel immunomodulatory agents and combination therapy that has changed the trajectory of cancer care. However, clinical benefit in patients with colorectal cancer has been generally limited to tumors with loss of mismatch repair function and those with specific germline mutations in the DNA polymerase gene. Unfortunately, tumors with these specific mutator phenotypes are in the minority. Recent pre-clinical and clinical studies have begun to reveal encouraging results suggesting that checkpoint inhibitor therapy can be expanded to an increasing number of colorectal tumors with microsatellite stability and the absence of traditional predictive biomarkers of checkpoint inhibitor response. These studies generally rely on combinations of checkpoint inhibitors with chemotherapy, molecular targeted therapy, radiation therapy, or other novel immunomodulatory agents. This article will review the most current data in microsatellite stable colorectal cancer.

## 1. Introduction

The dynamic interplay between tumor cells and the host immune system is instrumental in the evolution, progression and therapeutic susceptibility of colorectal cancer (CRC). Supporting the fundamental role of the immune system in the pathogenesis and course of this disease, colonic neoplastic progression is strongly associated with intestinal inflammatory mediators [1] and tumor-infiltrating lymphocytes (TILs) have been strongly implicated in the overall prognosis and clinical outcomes of this disease [2]. Nonetheless, despite the robust evidence for immune involvement in the development and progression of CRC, only with the successful application of immune checkpoint inhibitors (ICIs) in a distinct subset of CRC patients did the therapeutic potential and pharmacologic viability of modulating the inflammatory milieu of CRC cells clearly manifest. 

**Figure 1:**
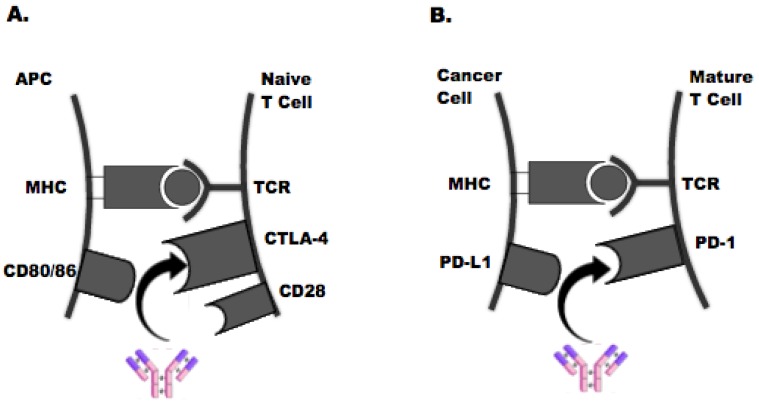
CTLA-4 and PD-1 Pathways. (**A**) This schematic depicts an antigen-presenting cell (APC) modulating early T-cell proliferation in the lymphatic system through CTLA-4 up-regulation. Following recognition and binding of a T-cell receptor (TCR) to a tumor-associated antigen expressed in the major histocompatibility complex (MHC), CTLA-4 outcompetes co-stimulatory receptor CD28 for binding to CD80/86 and dampens T-cell activation and proliferation. Reversal of this process with an antibody directed against CTLA-4 is shown. (**B**) This schematic shows a cancer cell regulating mature T-cell activation in peripheral tissue through PD-1 modulation. PD-1 (which can also be expressed on non-T cell subsets, including myeloid cells) interacts with its ligand PD-L1 on cancer cells or APCs to facilitate immune escape. Reversal of this process with a PD-1 antibody is depicted.

Tumor immune evasion is a fundamental hallmark of cancer cells [3], facilitated by T-cell “checkpoint” receptors such as cytotoxic T-lymphocyte-associated antigen-4 (CTLA-4) and programmed cell death protein-1 (PD-1) and is an important area of scientific investigation and interest (see Figure 1). Immune checkpoint inhibitors function by blocking the “checkpoint” receptors responsible for T-cell exhaustion and potentiate a robust tumor-directed immune response. Antibodies directed against PD-1, its ligand (PD-L1), and CTLA-4 have demonstrated remarkable efficacy in tumor types with a high mutational burden [4]. Mechanistically, experimental data suggest that increased tumoral mutational burden leads to the formation of immune-stimulating neoepitopes that directly boost tumor antigenicity. The subsequent recruitment of cytotoxic T-lymphocytes to the tumor microenvironment augments tumor immunogenicity and facilitates a more robust response to ICIs. As a validated predictive biomarker of ICI response in multiple tumor types, TILs also have been shown to have an independent prognostic role in the risk of CRC recurrence leading to the creation of an “immunoscore” to better classify the immune-features of these tumors [5]. 

For patients with hyper-mutated subtypes of CRC—specifically those with microsatellite instability-high (MSI-H)/mismatch repair-deficient (dMMR) CRC or those with hereditary mutations in the exonuclease domain of the polymerase epsilon, catalytic subunit (POLE)—ICIs have shown remarkable efficacy with a more favorable toxicity profile than traditional chemotherapy [6]. The notable success of these agents in MSI-H/dMMR CRC has been attributed to the favorable immunogenic features of these tumors that contrast starkly with the “immune-cold” features of microsatellite stable (MSS)/mismatch repair-proficient (pMMR) CRC. Specifically, dMMR CRC can yield about 20-times the amount of DNA mutations than tumors with pMMR, which fosters a more robust cytotoxic TIL response and improved ICI efficacy [7]. Likewise, in contrast to MSS CRC, MSI-H CRC is associated with up-regulated immune-related genetic and epigenetic signatures and increased expression of tumor PD-L1, which has been directly correlated with ICI response in multiple tumor types [8]. 

**Figure 2:**
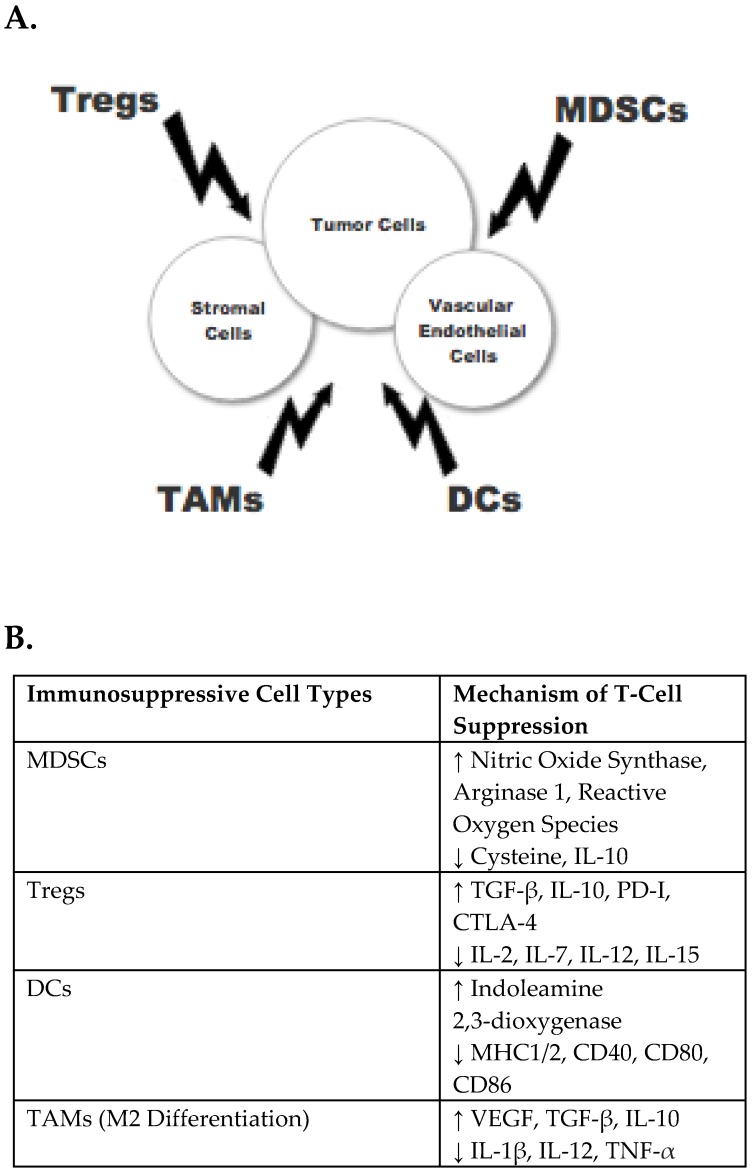
Cellular mediators of immune suppression in the tumor microenvironment. (**A**) Dysregulated immune cells that contribute to tumor immune escape include myeloid-derived suppressor cells (MDSCs), regulatory T-cells (Tregs), dendritic cells (DCs) and tumor-associated macrophages (TAMs). (**B**) Experimentally observed mechanisms of T-cell suppression by these cells are described, with multiple molecular mediators of immune suppression depicted. In addition, the cells have impaired tumorocidal activity and defective normal properties.

However, tumors with MSI constitute a small fraction of all colorectal neoplasms in both the metastatic and non-metastatic setting, and patients with the more prevalent MSS CRC have, on the whole, not benefited from single-agent ICI therapy. The exception is limited to roughly 3% of MSS CRC with a high tumor mutational burden and a more MSI-like phenotype [9]. Going forward, innovative therapeutic combinatorial approaches to sensitize “immuno-cold” MSS tumors to the activating effect of ICIs are currently under investigation, with the goal of surmounting intrinsic mechanisms of immune resistance that impair checkpoint monotherapy efficacy. Strategies involve overcoming the immunosuppressive tumor microenvironment (see Figure 2), boosting effector T-cell function, increasing tumor antigenicity, and up-regulating diverse molecular mediators to elicit a more “pro-inflammatory” tumor milieu. This review will explore the emerging ICI-based clinical trials, as well as the underlying preclinical rationale, driving new combination immune-based therapies in MSS CRC. 

## 2. Immune Checkpoint Inhibitors + Chemotherapy

In an attempt to foster a more immunogenic tumor microenvironment in MSS CRC, chemotherapy has been shown in vitro and in vivo to have favorable synergistic immunomodulatory effects that can potentiate ICI efficacy. As known predictive biomarkers critical to ICI effectiveness, TILs, tumor neoantigens, and tumor PD-L1 expression have been shown to increase after 5-flourouracil plus oxaliplatin (FOLFOX) treatment in murine models of CRC and human tumor sample correlates [10]. Likewise, a host of concurrent immunomodulatory effects—including upregulation of cellular major histocompatibility complex (MHC) class I, dendritic cell (DC) recruitment and activation, increased antigen presentation and suppression of regulatory T-cells (Tregs) and myeloid-derived suppressor cells (MDSCs)—have been reported post-chemotherapy and function synergistically in creating a more immune-promoting tumor microenvironment [11]. As a proof-of-principle, administration of oxaliplatin led to an increase in the overall immune response to a PD-L1 trap fusion protein in an in vivo murine pMMR CRC model [12].

Substantiated by these preclinical findings, results from a phase 3 trial of 559 patients with untreated, metastatic squamous non-small cell lung cancer (Keynote 407) showed an improved median overall survival (15.9 mo vs. 11.3 mo, *P* < 0.001) in patients receiving the PD-1 inhibitor pembrolizumab in combination with platinum-based chemotherapy first-line, irrespective of PD-L1 status [13]. With a notable superior survival advantage in the chemotherapy and ICI group, these early findings suggest that chemotherapy can augment ICI efficacy even in the absence of traditional biomarkers of response, with potential applicability for other tumor types, including MSS CRC. 

A host of current trials are underway in patients with MSS CRC to evaluate the utility of concurrent chemotherapy with checkpoint blockade. Preliminary results from a phase II study of FOLFOX followed by pembrolizumab in 30 patients with untreated, unresectable, and predominantly MSS CRC demonstrated an overall objective response rate (ORR) of 53% at 24 weeks median follow-up with a disease control rate (DCR) of 100% at 8 weeks [14]. Despite increased neutropenia in the initial 6 patient safety run-in, the large number of responses in this advanced, untreated cohort of pMMR CRC was clinically notable and worthy of further investigation. As such, additional chemotherapy combination regimens are under investigation in MSS CRC (please see Table 1) utilizing agents such as cytotoxan trifluridine with thymidine phosphorylase inhibitor tipiracil (TAS-102), histone deacetylase inhibitor romidepsin, DNA methyltransferase inhibitors 5-azacitidine and guadectiabine, and the folate antagonist pemetrexed. Additionally, a trial of locally-based trans-arterial tirapazamine embolization (TATE), a hypoxia-selective cytotoxan, in the context of metastatic CRC with liver lesions greater than 2 cm is currently recruiting patients. Notable co-administered ICIs include PD-1 inhibitors nivolumab and pembrolizumab, PD-L1 inhibitor durvalumab and CTLA-4 inhibitor tremelimumab among others. 

## 3. Immune Checkpoint Inhibitors + VEGF/EGFR Inhibitors +/− Chemotherapy

Validated as robust therapeutic targets in CRC, both the vascular endothelial growth factor (VEGF) and the epidermal growth factor receptor (EGFR) are well-established mediators of tumor growth and proliferation. Targeted agents directed against EGFR, such as cetuximab and panitumumab, and those directed against VEGF, such as bevacizumab, have been shown to facilitate a more immunogenic tumor profile in preclinical models and, as such, are reasonable potential adjuncts to ICIs in MSS CRC. In vitro and in vivo preclinical studies describe increased tumor necrosis receptor CD137 expression on NK and T-cells, decreased immunosuppressive cell populations (Tregs, MDSCs) and improved T-cell cytotoxicity and growth after EGFR inhibition [15]. Similarly, inhibition of VEGF has been shown in multiple studies to enhance immunity by decreasing immunosuppressive cell populations, increasing TILs and improving T-cell effector function [16]. Thus, the potential use of EGFR or VEGF inhibitors in conjunction with ICIs presents a promising strategy for treating MSS CRC. 

Driven by the preclinical data, an ongoing phase Ib/II study (NCT02713373) is evaluating the combination of cetuximab and pembrolizumab in patients with metastatic, RAS wild-type CRC with at least one prior line of treatment. In preliminary results of nine patients, the combination was well-tolerated despite the increased proportion of hypomagnesemia and led to durable (>16 weeks) disease control in six of the nine patients evaluated [17]. While more data are needed to better evaluate the efficacy and safety of this combination, a concurrent phase II study (NCT03442569) is evaluating nivolumab and ipilimumab with panitumumab in patients with metastatic, refractory, RAS wild-type, MSS CRC. 

Additional clinical strategies include adding the PD-L1 inhibitor atezolizumab to a backbone regimen of FOLFOX and bevacizumab. In the first-line metastatic CRC setting in 23 patients, these agents together safely yielded an ORR of 52% and a median progression-free survival (PFS) of 14.1 months with a median duration of response (MDR) of 11.4 months in a phase 1b study [18]. Supporting these clinical findings, tumor biopsies pre- and post- treatment demonstrated increased PD-L1 expression, cytotoxic T cell signatures and increased CD8+ T-cell proportions following the administration of both chemotherapy alone and in conjunction with VEGF inhibition. Likewise, a phase Ib open-label, multicenter study evaluating atezolizumab in combination with either bevacizumab alone in 14 metastatic, refractory CRC patients (arm A) or in combination with FOLFOX and bevacizumab in 30 oxaliplatin-naïve patients (arm B) reported an unconfirmed ORR of 8% in arm A and 36% in arm B at minimum follow-up of 1.9 months and 2.2 months, respectively [19]. Given the promising clinical activity noted, further studies investigating combining ICIs and anti-angiogenic agents are accruing data (see Table 2), with or without the concomitant use of cytotoxic chemotherapy. Selected agents include VEGFR2 inhibitor regorafenib, PD-L1 inhibitors atezolizumab and avelumab, PD-1 inhibitor PDR001 and CTLA-4 inhibitor ipilimumab among others.

## 4. Immune Checkpoint Inhibitors + Radiotherapy

Similar to chemotherapy and anti-angiogenic therapy, preclinical data support the use of radiotherapy as an effective means to augment ICI efficacy by directly contributing to the process of immunogenic cell death. Mechanistically validated in vivo and in vitro, radiotherapy can elicit a pro-inflammatory tumor state through direct tumor destruction, release of tumor antigens, up-regulation of inflammatory cytokines and cell surface molecules (i.e., MHC-1), and recruitment of immune cells into the tumor microenvironment [20]. The reported abscopal effect, in which tumors outside of the radiation treatment field can regress following radiation therapy, can be explained by a heightened systemic immune response induced by radiotherapy and supports the immune-promoting effects of this modality. 

Though early clinical efforts combining radiotherapy and ICI in other tumor types are limited, there are small trials showing potential benefit in the treatment of MSS CRC. Early data from a phase II, non-randomized study evaluating pembrolizumab 200 mg every 3 weeks in addition to either radiotherapy or ablation of tumor metastases in patients with advanced, refractory pMMR CRC reported 1 partial response in 11 patients (ORR 9%) who had received radiotherapy after a median of 4 doses of pembrolizumab [21]. No responses were noted in the ablation cohort. While more data are needed to fully gauge the true efficacy of this combination, this study suggests a generally tolerable toxicity profile associated with this approach. Fractionation optimization and proper sequencing and timing are required to better gauge the extent of the synergistic immune effect. Further information will be obtained from ongoing studies in CRC patients exploring the combination of radiotherapy and ICIs (please see Table 3). Radiotherapy strategies employed include local radioembolization to CRC metastases and external beam radiation therapy. 

## 5. Immune Checkpoint Inhibitors + MEK Inhibitors

As a key intermediate signaling molecule in the mitogen-activated protein kinase (MAPK) pathway, mitogen-activated protein kinase kinase (MEK) regulates cell cycle progression and is frequently aberrantly expressed in cancer cells [22]. Approximately 10% of metastatic CRCs have a mutation in the proto-oncogene BRAF upstream from MEK, frequently with a valine to glutamic acid change at codon 600 (V600E). These BRAF mutant tumors are commonly associated with sporadic MSI-H tumors and often exhibit a CpG island methylator phenotype. On the other hand, mutations in MAPK-regulator RAS, found in up to 60% of CRC patients, is more commonly found in MSS CRC and is resistant to EGFR-directed therapy [23]. Inhibitors of MEK, including trametinib, cobimetinib and binimetinib, which are commonly used in the treatment of BRAF mutated melanoma, have been shown in vivo and in vitro to have a number of positive immunomodulatory effects on the tumor microenvironment. Pre-clinical and human correlative studies have shown that MEK inhibition increases TILs and tumor-associated antigens, promotes the effector T-cell phenotype, and synergizes with and potentiates the effects of ICIs [24]. In addition, in vivo treatment of the C26 CRC cell line with dual PD-L1 and MEK inhibition increased suppression of tumor growth and led to more durable responses than did either agent alone, which can be explained through priming of the immune response by inhibition of MEK [25].

Clinical studies of ICIs and MEK inhibitors in CRC have been, on the whole, unremarkable to date. The 3-arm phase III randomized study IMblaze370 (NCT02788279) compared the combination of cobimetinib and atezolizumab with atezolizumab or regorafenib in 363 patients with metastatic, chemo-refractory, >90% MSS, CRC [26]. Neither atezolizumab monotherapy nor combination atezolizumab and cobimetinib demonstrated significantly improved OS compared to regorafenib in the intention-to-treat analysis. In a smaller phase Ib study of 84 chemo-refractory, heavily pre-treated, advanced CRC patients, combination atezolizumab and cobimetinib yielded seven confirmed partial responses, with four in patients that were not MSI-H, and a median OS of 10 months and a MDR of 14.8 months at the time of data cutoff [27]. The following is a table of ongoing studies testing these agents together (see Table 4).

## 6. Novel Combination Therapies

New actionable strategies to circumvent inherent MSS CRC immune resistance necessitate careful dissection of immune pathways and elements of the tumor microenvironment that pose barriers to checkpoint blockade. Unique pharmacologic approaches being studied in early-phase clinical trial are discussed below.

### 6.1. CSF1R Inhibitors

In vivo and in vitro experiments have demonstrated that growth suppression of tumor-associated macrophages (TAMs) through inhibition of colony-stimulating factor 1 receptor (CSF1R) can improve the anti-tumor, pro-inflammatory function of macrophages and enhance tumor immunogenicity for optimal ICI synergy [28]. While CSF1R-directed monotherapy has limited clinical efficacy data except in patients with diffuse-type teno-synovial giant cell tumors, adjunctive therapy with ICIs has shown evidence of early clinical efficacy in a small cohort of pancreatic cancer patients [29]. The utility of CSF1R inhibitors and ICIs in CRC, a tumor-type in which CSF1R-positive TAMs predominate and mediate an immunosuppressive tumor milieu [30], is currently under investigation in multiple early-phase studies (i.e., NCT02777710, NCT02452424, NCT02829723, NCT02880371).

### 6.2. IDO1 Inhibitors

Like CSFR1, the rate-limiting, cytosolic enzyme indoleamine 2,3-dioxygenase-1 (IDO1) functions in the process of tumor-mediated immune evasion. Inhibition of this enzyme, which has an essential role in regulating tryptophan catabolism, has been shown to improve tryptophan-dependent T-cell function, decrease the immunosuppressive product kynurenine, and reduce immunosuppressive tumoral cell populations [31]. Following pre-clinical experiments of demonstrated ICI/IDO1 synergy, a large phase 3 study of pembrolizumab in combination with the oral inhibitor of IDO1 (ECHO-301/KEYNOTE-252), epacadostat, failed to show benefit in patients with melanoma compared to pembrolizumab alone. However, while the results were disappointing in melanoma, the combination is still under investigation in patients with CRC. Ongoing studies are evaluating these two agents in patients with solid tumors, including CRC, alone (NCT02880371) and in combination with azacitidine (NCT03182894). 

### 6.3. Autologous Tumor Vaccines and Oncolytic Viral Therapy

Therapeutic cancer vaccines represent an alternative strategy for immune-sensitization that may be rationally combined with ICIs to augment anti-tumor immunity. In particular, the GVAX CRC vaccine, which consists of irradiated CRC cells modified to express granulocyte-macrophage colony-stimulating factor (GM-CSF), is currently being investigated in combination with cyclophosphamide and pembrolizumab in patients with pMMR advanced CRC (NCT02981524). Likewise, the adenovirus carcinoembryonic antigen (ad-CEA) vaccine, designed to procure a CEA-specific immune response, is also being evaluated in combination with PD-L1 inhibition in patients with metastatic or unresectable CRC (NCT03050814). An alternative personalized peptide vaccine, which combines individually selected, human leukocyte antigen (HLA)-matched peptides derived from a panel of tumor-associated antigens, is also being trialed with PD-1 inhibition (NCT02600949). Similarly, a personalized neoantigen vaccine GRT-C901/GRT-R902 is being evaluated in combination with nivolumab and ipilimumab in patients with MSS CRC (NCT03639714). 

In contrast, oncolytic viral therapy mediates tumor regression through preferentially replicating and destroying tumor cells, leading to a proliferative immune cascade. Talimogene Laherparepvec (T-vec) was the first oncolytic virus approved by the US Food and Drug Administration in 2015. It is a modified herpes simplex virus (HSV) type 1 with an inserted GM-CSF gene delivered via intralesional injection that selectively replicates in and lyses melanoma cells. In a phase III trial, T-vec improved durable response rate and ORRs compared to subcutaneous GM-CSF, although the improvement in OS only trended to statistical significance [32]. The success of this oncolytic virus was the culmination of significant advances in the understanding of tumor biology, immunity, and genetic manipulation. Anti-tumor activity of oncolytic viruses can occur by direct viral replication as well as induction of specific and non-specific anti-tumor immunity. A variety of viral platforms, including vaccinia virus, HSV, and adenovirus are being evaluated as single agents in CRC clinical trials [33]. Approaches combining oncolytic viruses with additional immuno-oncology approaches such as ICIs and cellular immunotherapy are ongoing [34]. Recently, a randomized phase II trial of FOLFOX plus bevacizumab with or without pelareorep, an oncolytic reovirus that replicates in RAS mutated cells, in patients with metastatic CRC noted that the study cohort had a statistically increased ORR but also a statistically reduced PFS and duration of response [35]. These mixed results reveal the hurdles that oncolytic viral therapy must still overcome. Some of these challenges include optimizing tumor tropism, viral delivery, and enhancing anti-tumor immunity [36]. Intralesional injection can produce an abscopal effect in untreated lesions, but overcoming potential mechanisms of viral inactivation when administered intravenously is still appealing. Oncolytic viral cell death can induce an adaptive immune response, raising the possibility that oncolytic viruses can transform a MSS CRC into a target of the immune response [37]. 

### 6.4. T-Cell Bispecific Antibodies

The use of bioengineered T-cell bispecific antibodies (TCBs) with the ability to simultaneously target and connect both a tumor associated antigen and a CD3 T-cell receptor epitope directly enables a localized, antigen-specific T-cell response. Early pre-clinical and clinical data suggest that TCBs against the carcinoembryonic antigen (CEA), which are a known CRC biomarker, can lead to up-regulated expression of PD-L1, increase intra-tumoral lymphocytes and augment anti-tumor activity [38]. The combination of TCB-CEA with atezolizumab is currently accruing data in CEA-positive tumors, including CRC (NCT02650713). Likewise, similar approaches involving TCBs against other tumor-associated antigens, including the cell surface glycoprotein A33, especially prevalent in CRCs, is in a clinical trial with ICIs (NCT03531632).

### 6.5. CD73 Inhibitors

Adenosine monophosphate is dephosphorylated to adenosine by the extracellular ectonucleotidase CD73, which is highly expressed on the plasma membrane of cancer and immune cells [39] Adenosine can then bind with the adenosine A2A receptor (A2AR) to exert immunosuppressive effects on the tumor microenvironment. Therefore, inhibition of this enzyme has been a proposed treatment strategy for creating a more pro-inflammatory tumor microenvironment conductive to ICI synergy. The use of this agent in CRC stems from preclinical experiments showing CD73′s role in the proliferation of colonic tumerogenisis and its high expression level in these tumors [40]. Current clinical trials with ICIs and anti-CD73 antibodies (NCT02503774), A2AR antagonists (NCT03207867) and combinations of both (NCT03549000) are accruing data.

### 6.6. Gut Microbiota

The gut microbiome has an important role in regulating the innate and adaptive immune response in the presence of foreign pathogens. Specific subpopulations of intestinal microbes have been implicated in the efficacy of ICIs [41]. Altering the intestinal and intratumoral microbiota using a lipopolysaccharide (LPS)-targeting fusion protein directed against CRC is a potential novel strategy to boost host immunity and PD-L1 efficacy and has shown potential promise in early studies [42]. LPSs, which are prevalent in human CRC tissue, are large glycolipids found on the outer-membrane of gram-negative bacteria and have a demonstrated association with ICI inactivity in CRC. 

### 6.7. Poly-ICLC 

Polyinosinic-polycytidylic acid-poly-l-lysine carboxymethylcellulose (poly-ICLC) is a ligand for toll-like receptor 3 and has a critical role in stimulating the systemic innate immune response [43]. Through interferon-directed up-regulation of PD-L1 and reactive recruitment of TILs, this treatment is thought to complement the immune-activating effect of ICIs in CRC. A phase I/II trial of pembrolizumab and poly-ICLC in patients with metastatic pMMR CRC is currently recruiting (NCT02834052).

### 6.8. Additional Investigative Agents

Please see Table 5 for additional therapeutic agents with a broad range of diverse molecular targets currently being evaluated in combination with ICIs in MSS CRC. Drugs directed at chemokines, or small pro-inflammatory cytokines, and their receptors, are intended to selectively modulate the immune features of these molecules, which have an important role in maintaining tissue homeostasis and immune cell activity in the tumor microenvironment [44,45]. Specific inhibitors of the receptor CXCR2 (navarixin), the chemokine CXCL12 (olaptesed pegol) and the receptor CCR5 (vicriviroc) are accruing data in combination with ICIs. Additional mediators of tumor immune cell activity in CRC cells include heat shock protein 90 (HSP90), for which the inhibitor XL888 is being combined with pembrolizumab, as well as Bruton’s tyrosine kinase (BTK) on MDSCs, for which the BTK-inhibitor ibrutinib is being combined with pembrolizumab in MSS CRC [46,47]. 

Therapeutics targeting well-delineated molecular pathways with known roles in tumor immunity offer alternative agents for ICI combination therapy in MSS CRC. The WNT/ β-catenin signaling pathway—frequently mutated in MSI-H tumors and associated with reduced TIL density in CRCs [48]—can be inhibited via the small-molecule porcupine (PORCN) inhibitor CGX1321 [49] or the STAT3/WNT inhibitor BBI608 [50]. Likewise, inhibitors of the phosphoinositide 3-kinase (PI3K) pathway (copanlisib) and the MAPK/ERK pathway (MK 8353, eFT508), both important regulators of cancer progression and the tumor immune microenvironment, are in clinical trial with ICIs. Lastly, immune stimulants, such as prostaglandin E receptor (EP4) antagonist grapiprant and the novel checkpoint inhibitor of lymphocyte activation gene-3 (LAG-3) are being combined with PD-1 inhibitors. While chimeric antigen receptor T-cell (CAR-T) therapy has been successful in hematologic malignancy, at the time, current scientific barriers limit its efficacy in solid tumors, though it may hold promise in the future. 

## 7. Conclusions

ICIs that target either PD-1 or PD-L1 can have remarkable efficacy in any MSI-H malignancy, including MSI-H CRCs. No clinical differences in efficacy have been detected between these two antibody targets, although PD-1 blockade has been utilized in the majority of CRC studies. With the clinical success of ICIs in the MSI-H population, significant effort is now being deployed to harness the benefits of immune therapy in the MSS CRCs. Combination strategies have shown promise with the administration of ICIs with a variety of additional agents, including cytotoxic chemotherapy, small molecule inhibitors, targeted agents against the MAPK pathway, and radiotherapy techniques. Dual checkpoint inhibition of PD-1 plus CTLA-4 has been approved in MSI-H CRCs but there no reported clinical trials in MSS patients. Additional adjunctive agents under investigation include CSFR1 inhibitors, IDO1 inhibitors, TCBs and a host of other agents. As these novel agents progress through the phases of clinical development, novel predictive markers specific to these agents may become important to optimally select patients most likely to benefit from them. Going forward, more data are needed to address efficacy and tolerability as well as drug sequencing, dosing, and timing to optimize patient benefit. Careful consideration will need to be given to the cost of these medications in the overall paradigm of CRC treatment. 

## Figures and Tables

**Table 1:** Selected clinical trials of immune checkpoint inhibitors (ICIs) with chemotherapy in patients with colorectal cancer (CRC).

Combination Treatment	ICI	Study Population	Trial ID	Phase	Status
Trifluridine + Tipiracil Hychloride	Nivolumab	Refractory, Metastatic MSS CRC	NCT02860546	II	Completed
Romidepsin +/− 5-Azacitidine	Pembrolizumab	Refractory, Metastatic MSS CRC	NCT02512172	I	Recruiting
Pemetrexed +/− Oxaliplatin	Pembrolizumab	Refractory, Metastatic MSS CRC	NCT03626922	I	Not yet Recruiting
Nordic FLOX Regimen	Nivolumab	Untreated, Metastatic MSS CRC	NCT03388190	II	Recruiting
Azacitidine	Durvalumab	Refractory, Metastatic MSS CRC	NCT02811497	II	Recruiting
Guadecitabine	Nivolumab	Refractory, Metastatic MSS CRC	NCT03576963	Ib/II	Not yet Recruiting
FOLFOX	Tremelimumab + Durvalumab	First-line, KRAS-mt CRC	NCT03202758	Ib/II	Recruiting
TATE	Nivolumab or Pembrolizumab	Metastatic CRC to liver	NCT03259867	II	Recruiting

Abbreviations: mt, mutant; MSS, microsatellite stable; FOLFLOX, 5-flourouracil plus oxaliplatin; TATE, trans-arterial tirapazamine embolization; FLOX, 5-flourouracil, folinic acid and oxaliplatin.

**Table 2:** Selected clinical trials of ICIs in combination with molecularly targeted agents in patients with CRC.

Combination Regimen	ICI	Study Population	Trial ID	Phase	Status
Capecitabine + Bevacizumab	Atezolizumab	Refractory, Metastatic CRC	NCT02873195	II	Not Recruiting
SOC Chemotherapy + Bevacizumab	Nivolumab	Metastatic CRC; No Prior Chemotherapy	NCT03414983	II/III	Recruiting
Trifluridine/Tipiracil + Oxaliplatin +/− bevacizumab	Nivolumab	Refractory, Metastatic CRC	NCT02848443	I	Recruiting
Regorafenib	PDR001	Refractory, Metastatic MSS CRC	NCT03081494	I	Recruiting
Capecitabine + Bevacizumab	Pembrolizumab	Refractory, MSS CRC	NCT03396926	II	Recruiting
Cetuximab + Irinotecan	Avelumab	Refractory, BRAF V600E-WT, MSS CRC	NCT03608046	II	Not yet Recruiting
Bevacizumab + mFOLFOX6	PDR001	Treatment naïve, MSS CRC	NCT03176264	Ib	Completed
Panitumumab	Nivolumab + Ipilimumab	Refractory, KRAS/NRAS/BRAF-WT, MSS CRC	NCT03442569	II	Recruiting

Abbreviations: SOC, Standard-of-Care; WT, wild-type.

**Table 3:** Clinical trials of combination ICIs and radiotherapy in CRC patients.

Radiation Regimen	ICI	Study Population	Study ID	Phase	Status
Standard Radiation Therapy	Nivolumab+ Ipilimumab	MSS and MSI-H CRC	NCT03104439	II	Recruiting
Hypofractionated palliative radiation	Durvalumab and Tremelimumab	Metastatic MSS CRC	NCT03007407	II	Recruiting
Chemo-radiation	Durvalumab	Stage II-IV, MSS Rectal Cancer	NCT03102047	II	Recruiting
SBRT to Liver	Pembrolizumab	Metastatic CRC to Liver	NCT02837263	I	Recruiting
Radioembolization	Durvalumab and Tremelimumab	Metastatic MSS CRC to Liver	NCT03005002	I	Active, not recruiting
High or low-dose radiation therapy	Durvalumab and Tremelimumab	Refractory Metastatic MSS CRC to Liver	NCT02888743	II	Recruiting

Abbreviations: SBRT, stereotactic body radiation therapy.

**Table 4:** Selected studies of combination ICIs and MEK inhibitors in CRC.

Combination Regimen	ICI	Study Population	Study ID	Phase	Status
Trametinib	Nivolumab +/− Ipilimumab	RAS-mt; previously treated, metastatic MSS CRC	NCT03377361	I/II	Recruiting
Binimetinib	Nivolumab +/− Ipilimumab	RAS-mt; previously treated, metastatic MSS CRC	NCT03271047	I/II	Not Recruiting
Dabrafenib + Trametinib	PDR001	BRAFV600E-mt; metastatic CRC	NCT03668431	II	Recruiting
Trametinib	Durvalumab	Refractory, metastatic MSS CRC	NCT03428126	II	By Invitation

**Table 5:** Novel combination therapies in MSS CRC.

Novel Agents	Therapeutic Targets	ICI	Study ID
Navarixin	CXCR2	Pembrolizumab	NCT03473925
Olaptesed Pegol	CXCL12	Pembrolizumab	NCT03168139
eFT508	MNK 1/2	Avelumab	NCT03258398
Ibrutinib	BTK	Pembrolizumab	NCT03332498
XL888	HSP	Pembrolizumab	NCT03095781
CGX1321	PORCN	Pembrolizumab	NCT02675946
BBI608	STAT3/WNT	Pembrolizumab	NCT02851004 NCT03647839
Vicriviroc	CCR5	Pembrolizumab	NCT03631407, NCT03274804
Grapiprant	EP4	Pembrolizumab	NCT03658772
Relatlimab	LAG-3	Nivolumab	NCT03642067
Copanlisib	PI3K	Nivolumab	NCT03711058
MK-8353	ERK1/2	Pembrolizumab	NCT02972034
